# Universality of fragment shapes

**DOI:** 10.1038/srep09147

**Published:** 2015-03-16

**Authors:** Gábor Domokos, Ferenc Kun, András Árpád Sipos, Tímea Szabó

**Affiliations:** 1 Department of Mechanics, Materials and Structures, Budapest University of Technology and Economics, Műegyetem rkp. 3., K242, 1111 Budapest, Hungary; 2 Department of Theoretical Physics, University of Debrecen, H-4010 Debrecen, P.O.Box: 5, Hungary

## Abstract

The shape of fragments generated by the breakup of solids is central to a wide variety of problems ranging from the geomorphic evolution of boulders to the accumulation of space debris orbiting Earth. Although the statistics of the mass of fragments has been found to show a universal scaling behavior, the comprehensive characterization of fragment shapes still remained a fundamental challenge. We performed a thorough experimental study of the problem fragmenting various types of materials by slowly proceeding weathering and by rapid breakup due to explosion and hammering. We demonstrate that the shape of fragments obeys an astonishing universality having the same generic evolution with the fragment size irrespective of materials details and loading conditions. There exists a cutoff size below which fragments have an isotropic shape, however, as the size increases an exponential convergence is obtained to a unique elongated form. We show that a discrete stochastic model of fragmentation reproduces both the size and shape of fragments tuning only a single parameter which strengthens the general validity of the scaling laws. The dependence of the probability of the crack plan orientation on the linear extension of fragments proved to be essential for the shape selection mechanism.

Fragmentation of solids into numerous pieces occurs on widely different time scales ranging from the geomorphic evolution shaping landforms to the energetic breakup of solids used in mining and ore processing^1^,^2^,^3^,^4^,^5^,^6^. Slowly growing cracks due to weathering give rise to the spallation of pieces from the surface of rock walls^6^,^7^,^8^,^9^,^10^,^11^. At the opposite limit instantaneous breakup occurs when a large amount of energy is imparted to a solid within a short time, e.g. by explosion or impact^12^,^13^,^14^,^15^,^16^,^17^,^18^,^19^,^20^,^21^,^22^,^23^,^24^,^25^. At intermediate time scales the gradual size reduction of solid bodies observed for diverse systems such as aerosol particles in the atmosphere^26^,^27^, pebbles in river beds^8^,^9^,^10^, or asteroids in the Solar system^28^, is the consequence of repeated sub-critical collisions.

The shape of fragments plays a central role for the understanding of a broad class of phenomena having also practical importance. Figure 1 demonstrates for a piece of rock obtained in an explosion experiment that the geometrical structure of fragments has a high degree of complexity from the lowest length scale of the surface texture to the overall shape of the object. The shape of rocks in mountain boulder fields and of pebbles in river beds observed today are all results of a long evolution of abrasion and spallation processes^1^,^2^,^3^,^8^,^9^,^10^. The initial configuration of these types of geomorphic evolution is freshly fractured rock like in Fig. 1 which is mainly generated by energetic fragmentation processes occurring in earthquakes, volcanic eruptions, cliff collapses, landslides, and stone avalanches. Recent discoveries of traces of fluvial evolution of landforms on planet Mars such as cemented conglomerates of pebbles underlines the importance of understanding their origin^29^. A special area of human activity where fragmentation phenomena play an extraordinary role is space exploration which has led to the accumulation of space debris over the past decades. The main source of space debris is the impact or explosion induced breakup of rocket components and unused satellites orbiting Earth^30^. To estimate the lifespan of space debris and to asses the risk they present for satellites and manned space missions knowledge on the shape of fragments is essential^30^,^31^.

During the past decades research on fragmentation mainly focused on the statistics of fragment masses *m* (or sizes) which revealed power law distributions
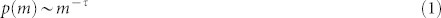
with a high degree of robustness of the exponent *τ* against materials' details and loading conditions^5^,^6^,^12^,^14^,^23^,^25^. The value of the exponent *τ* is mainly determined by the dimensionality of the system^14^,^15^,^16^,^17^,^18^,^19^,^20^,^21^,^22^,^23^,^25^,^32^,^34^,^35^ and by the brittle and ductile character of the mechanical response of the material involved^33^. Here we present a thorough experimental and theoretical study of the shape of fragments originating from slowly evolving weathering and from rapid breakup induced by explosion and hammering. Based on field measurements and laboratory experiments we demonstrate that the shape of fragments of heterogeneous brittle materials have universal characteristics. We go beyond the overall description of fragments and explore the richness of fragment shapes by analyzing the statistics of their stable and unstable static equilibria. We introduce a simple stochastic model of breakup which reproduces all the observed scaling laws of fragment shapes emphasizing the robustness of the results and revealing the mechanism of shape selection.

## Results

### Fragmentation experiments

In order to understand the shape selection mechanism of fragments, we performed seven sets of experiments considering two cracking mechanisms which give rise to fragment formation: Weathering induced slow growth of cracks gradually removes pieces from the surface of rock walls under the action of gravity. Such surface fragments were populated either by collecting them from the foot of a cliff where they accumulated (experiment A), or by removing them from the mother rock by gently hammering the surface. The hammering experiments were repeated with limestone and dolomite at different geographical locations (experiments B, C, D,). Explosion experiments were performed in stone mines generating a rapid breakup of a rock wall into a large number of pieces (experiments E, F). Field measurements on geomaterials were complemented by laboratory experiments fragmenting gypsum cubes by manual hammering applied in a dynamic way (experiment G). Table 1 summarizes the most important parameters of the experiments, further details are presented in Methods. The number of fragments in the experiments was ranging from a few hundred to one thousand, providing reasonable statistics for the data evaluation. The maximal size *L* of the collected fragments was in the range 15 mm < *L* < 150 mm and we attempted to collect all available fragments in this range in a given area. For the quantitative characterization of the fragment ensembles we determined the mass of single pieces and characteristic quantities of the shape of fragments.

The probability distribution of the mass of fragments *p*(*m*) is one of the most important characteristics of fragmentation phenomena^1^,^2^,^5^,^12^,^14^,^15^,^16^,^17^,^18^,^19^,^20^,^21^. Figure 2 demonstrates that for all sets of experiments *p*(*m*) has a power law functional form described by equation (1). The sampling method applied when collecting fragments implies cutoffs both for low and high mass values but in the intermediate mass regime of the distributions the power law form prevails in all cases. A very important feature of the results is that the distributions *p*(*m*) are described by a unique exponent *τ* = 1.7 ± 0.06 in spite of the strongly different cracking mechanisms governing fragment formation in dynamic fragmentation initiated by explosion and hammering and in weathering induced spallation. The value of the exponent *τ* falls close to the analytic prediction of the branching-merging scenario of dynamic cracks in three dimensions^23^,^34^,^35^: cracks propagating at a high speed undergo sequential branching and fragments are formed as material regions enclosed when sub-branches merge^34^,^35^. Assuming the self-similar nature of the developing crack-tree, power law distributed fragment masses are obtained with a universal exponent *τ* = (2*D* − 1)/*D* depending solely on the dimensionality *D* of the system. For three-dimensional bulk solids *τ* = 5/3 follows in a good agreement with our experimental findings. It is astonishing that the same universality class is recovered when the fragment population is generated by weathering (experiments A, B, C, and D). The result can be explained by the fact that environmental aging typically gives rise to the growth of pre-existing cracks instead of creating new ones. Cracks slowly advance due to thermal expansion, hydration, frost induced expansion, etc, gradually removing pieces from the surface. Hence, the agreement with the prediction of the branching-merging scenario may imply that during their geological history the rocks considered have experienced dynamic loading.

**Figure 1:**
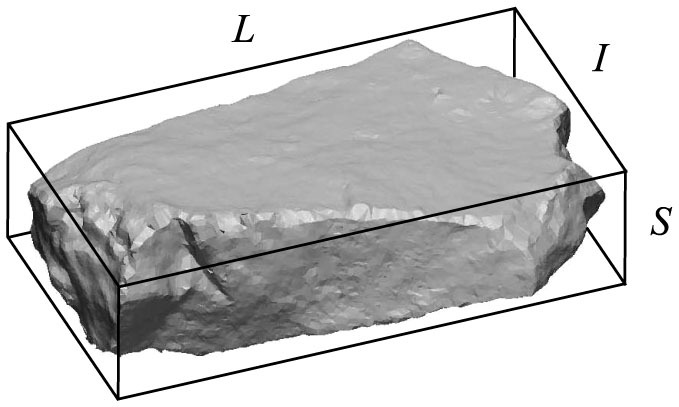
An example of a rock fragment generated by an explosion in a stone mine. The three-dimensional image was obtained by scanning the sample. The wire-frame around the fragment represents the bounding box with linear extensions *S* < *I* < *L*.

From the geometrical point of view, freshly created fragments are polyhedra with sharp edges and corners as it is illustrated in Fig. 1. As a first step we focus on the overall shape of rock pieces neglecting any surface texture created by the cracking mechanism. Later on the analysis will be refined by considering details of polyhedral shapes. In order to define an appropriate shape descriptor we construct the bounding box of each fragment with principal axis *S* < *I* < *L* (see *Methods* for details). The value of *L* provides a measure of the linear extension of fragments, while the dimensionless ratios *S*/*L* and *I*/*L*, and their relations characterize the shape of pieces^11^,^36^,^37^,^38^,^39^,^41^,^42^,^43^.

Figure 3 presents the value of the shape parameters *S*/*L* and *I*/*L* as function of the fragment size *L*. The most remarkable feature of the results is that for all materials and fragmentation modes the same shape-size relation is obtained: for small values of *L* the shape parameters *S*/*L* and *I*/*L* rapidly approach one which implies that small sized fragments have an isotropic shape with *S* ≈ *I* ≈ *L*. As the fragment size *L* increases both *S*/*L* and *I*/*L* decrease and tend to finite constant values *B_S_* and *B_I_*, respectively. The size dependence of fragment shape in Fig. 3 can be well described by an exponential form
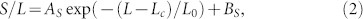

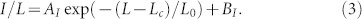
Here the cutoff length *L_c_* provides the fragment size where the isotropic shape is reached, while the scale parameter *L*_0_ controls how fast the converges is to the unique shape of large fragments. Equations (2,3) also indicate that the characteristic length scales *L*_0_ and *L_c_* have the same values for *S* and *I*. In Fig. 3 best fit was obtained with the parameter values *A_S_* = 0.45, *A_I_* = 0.4, *B_S_* = 0.43, *B_I_* = 0.67, *L*_0_ = 7.2 mm, and *L_c_* = 17.0 mm. The results imply that large enough fragments *L* ≫ *L_c_* + *L*_0_ have a unique elongated shape characterized by the same value of the shape parameters *S*/*L* ≈ *B_S_* and *I*/*L* ≈ *B_I_*.

Besides the shape-size relation of fragments the statistics of the occurrence of different shapes in a fragment ensemble is also of fundamental importance. The breakup models of NASA use the surface-to-volume ratio *A*/*V* to characterize the shape of objects generated by on-orbit fragmentation events^30^,^31^. In order not to have dependence on the extension of objects, following Ref. 32, we multiply this combination with the radius of gyration *R_g_* and define the shape parameter as *S_f_* = *AR_g_*/*V*. Assuming rectangular shape of fragments *R_g_* reads as 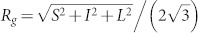 and *S_f_* can be cast into the form 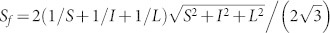. For cubic fragments *S* ≈ *I* ≈ *L* the shape parameter simplifies to *S_f_* = 3 independent of the size of fragments, while larger values *S_f_* > 3 characterize elongated shapes. To quantify the statistics of occurrence of different shapes in fragment populations, we determined the probability distribution *p*(*S_f_*) of the shape parameter. In Fig. 4 again a robust behavior is obtained, i.e. in all experiments *p*(*S_f_*) exhibits an exponential decay
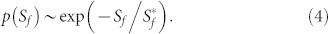
The scale parameter 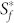 proved to have a relatively low value 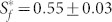 which shows that the contribution of anisotropic shapes provides a considerable fraction to the fragment ensemble.

The dimensionless ratios *S*/*L*, *I*/*L*, as well as, the shape parameter *S_f_* carry rather limited information on the geometry of the fragment: for example, consider that at *any* fixed values for these parameters the fragment could be either ellipsoidal, tetrahedral, cuboid or even octahedral. To distinguish between these (and other) geometries with identical side ratios, we use the numbers *n_S_* and *n_U_* of stable and unstable static equilibrium points^44^ which can be counted in simple hand experiments and have been already used to assess geological data^45^. In the previous example, for ellipsoids, tetrahedra, cuboids and octahedra we get *n_S_* = 2, 4, 6, 8 and *n_U_* = 2, 4, 8, 6, respectively. The definition of stable and unstable equilibria is illustrated in Supplementary Fig. S1. Figure 5 presents the probability distributions of the number of stable *p*(*n_S_*) and unstable *p*(*n_U_*) points of fragments for all experiments. The remarkable result is that both *n_S_* and *n_U_* scatter over a broad range with a common minimum value of 2 and maxima reaching to *n_S_* = 9 and *n_U_* = 11, which demonstrate the richness of shapes not captured by other approaches. The data can be well fitted by a log-normal form with parameters *µ* = 1.54, *σ* = 0.22, and *µ* = 1.63, *σ* = 0.29, for the stable and unstable equilibrium points, respectively. (For details of the functional form of the distributions *p*(*n_S_*) and *p*(*n_U_*) see Supplementary Information.)

### Discrete stochastic model of fragmentation

**Figure 2:**
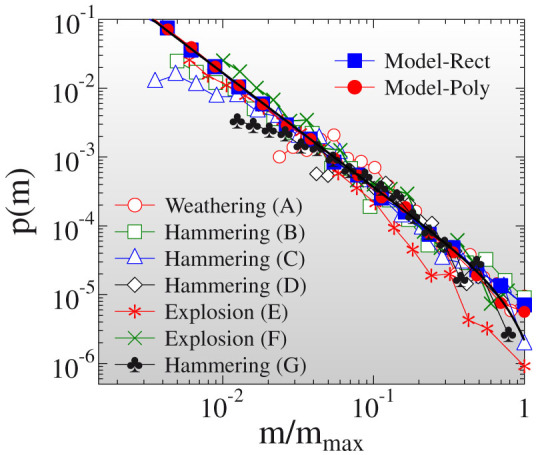
Mass distribution of fragments *p*(*m*) obtained by experiments and by computer simulations. The mass *m* is normalized by the largest fragment mass *m_max_* of the data sets. The legend indicates how the fragment populations were generated, for more details of the experiments see Table 1 and *Methods*. The data sets were arbitrarily rescaled along the vertical axis to obtain collapse of the distributions. The bold line represents best fit with a power law followed by an exponential cutoff.

In order to understand how the disorder of materials and the dynamics of disruption leads to the emergence of the observed scaling laws of fragment shapes, we constructed a generic stochastic model of fragmentation (Model-Rect). The model is based on a sequential binary breakup of fragments^1^,^2^,^6^,^32^,^40^ starting from a single body which has an initial cubic shape. At each step of the hierarchy fragments either break into two pieces of equal mass with probability 0 < *p* < 1 or they keep their current size until the end of the process with probability 1 − *p*. Iterating the stochastic dynamics for all active pieces a power law mass distribution of fragments is obtained. The model was calibrated by setting the value of the breaking probability to *p* = 0.8 which provides a very good agreement with the measured mass distribution of fragments in Fig. 2 (see also Methods and Supplementary Information).

**Figure 3:**
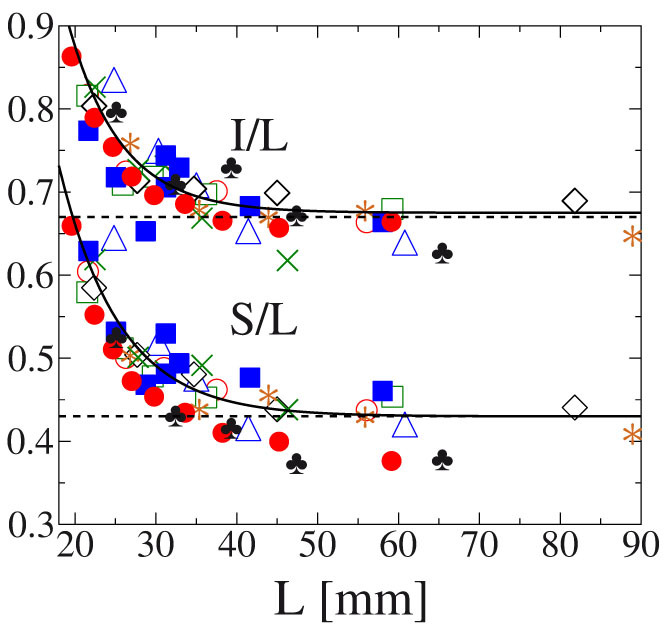
The dimensionless ratios *S*/*L* and *I*/*L* of the principal axis of fragments as a function of the value of the largest axis *L*. (The legend is the same as in Fig. 2.) For small fragment sizes both quantities approach one while for increasing *L* they decrease and converge to finite constant values indicated by the dashed horizontal lines. The bold lines represent fits with equations (2,3). Results of the model calculations are in a good agreement with the experimental findings.

**Figure 4:**
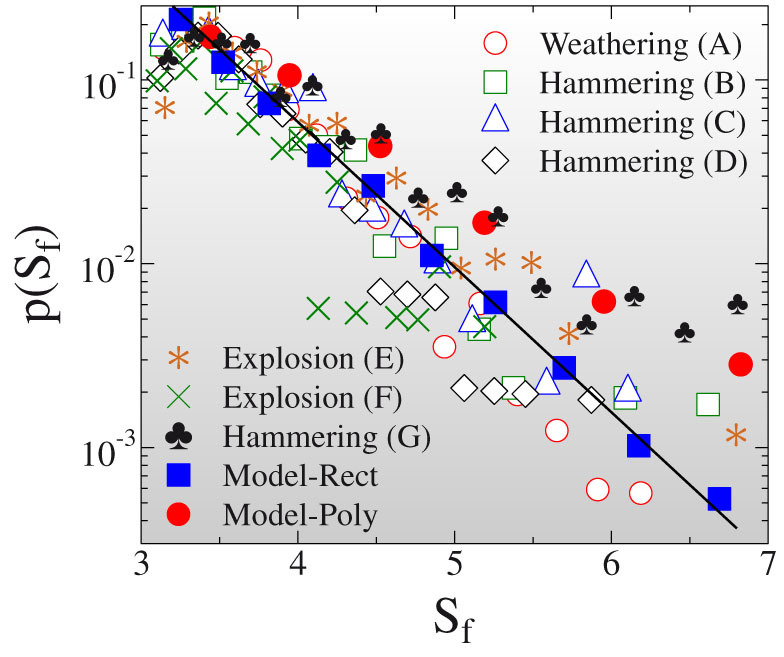
Probability distribution of the shape parameter *S_f_* on semilogarithmic plot. The straight line represents a fit with an exponential function. Both Model-Rect and Model-Poly reproduce the exponential functional form of p(*S_f_*).

To follow how the shape of pieces evolves, we assume that cracks always occur in the middle of edges cutting through the center of mass *C* of the body. Figure 6 illustrates that for any fragment (Fig. 6(a)) there are three possibilities for crack formation (Figs. 6(b, c, d)), which all result in two pieces of equal mass. (This breakup mechanism is in spirit the three-dimensional generalization of the fragmentation model of Refs. 41, 42.) To capture the effect that it is easier to break a body perpendicular to a longer extension, we choose among the three possibilities with a probability *p_c_* proportional to a power *α* of the length of the breaking edge *l*
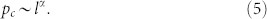
This cracking mechanism retains the orthogonal form of pieces and gives rise to a complex dynamics of shape selection controlled by the exponent *α*, i.e. *α* = 0 implies completely random cracking while *α* > 0 favors the breaking of longer edges. Figures 3 and 4 demonstrate that setting *α* = 3 for the exponent of *p_c_* an excellent agreement is obtained with the experimental findings. The model reproduces the exponential convergence from the isotropic form of small fragments to a unique anisotropic shape of the large ones (Fig. 3), and it recovers the exponential distribution of the occurrence of different shapes (Fig. 4), as well.

In the case of orthogonal fragments (cuboids) considered above *n_S_* and *n_U_* have the values *n_S_* = 6 and *n_U_* = 8 which remain constant as the breakup proceeds so that this simple model cannot account for the geometric complexity encoded in the statistics of equilibria. To extend the model beyond orthogonal shapes we let the crack plane orientation vary continuously: the crack always crosses the center of mass of fragments, however, its normal vector *n* can take any direction according to a continuous probability distribution of the form of equation (5). In the model calculations the linear extension *l* of fragments is measured from the center of mass *C* and we create a list 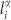 based on these distances from which our stochastic algorithm can uniformly sample. Each linear extension *l_i_* is associated with a unit vector *n_i_*. In case of a polyhedron *P* with *F* faces and *V* vertices we have several alternatives to create this list, out of which we mention three:

  (a) the list *l*_(*f*),*i*_ (*i* = 1, 2, … *F*) of the distances of planar faces, the associated unit vectors are the unit normals of the given face.

  (b) the list *l*_(*v*),*i*_ (*i* = 1, 2, … *V*) of the distances of the vertices, the corresponding unit vectors are collinear with these lines.

  (c) the list *l*_(*r*),*i*_ (*i* = 1, 2, … *N*) associated with *N* tangent planes, the orientations 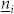 of which are uniformly random on the unit sphere.

The different definitions of the lists of length are illustrated in Supplementary Fig. S2. We remark that list (*c*) is closely associated with the support function of *P*^47^. It is relatively easy to see that while (*a*) underestimates the largest linear extension, (*b*) overestimates it and only (*c*) appears to be unbiased. For each type of list we numerically computed the *α* exponent providing the best fit to the experimental data, and in perfect agreement with the above observation we found that in the three cases we get *α* ≈ 3, *α* ≈ 0.2 and *α* ≈ 1, respectively. Nevertheless, list (*a*) is a natural choice for the cuboid model (Model-Rect) so we used it in those computations. In case of the polyhedral model (Model-Poly) we used the (*c*)-list. This shows that the physically relevant value of the exponent is *α* = 1, while other values merely compensate for the bias in the definition of the linear extension. Hence, when fragment shapes are realistically captured the parameter *α* can be removed from the model.

**Figure 5:**
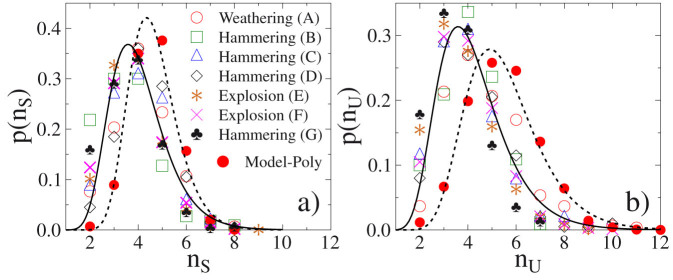
Probability distribution of the number of stable (*a*) and unstable (*b*) equilibrium points of fragments. The extended discrete model of fragmentation (*Model*-*Poly*) is able to reproduce the measured statistics of equilibria with a reasonable precision. Both the experimental and theoretical results are fitted with a lognormal distribution represented by the continuous and dashed lines, respectively.

**Figure 6:**
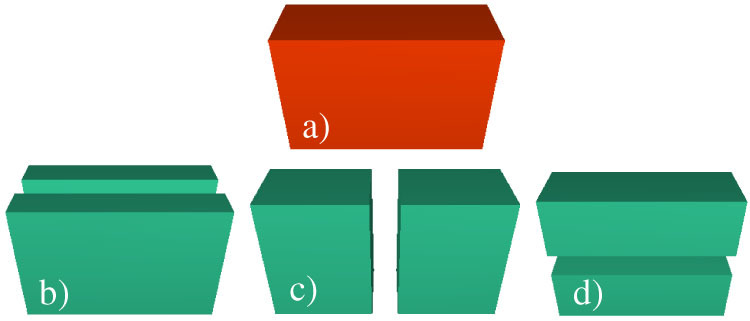
Breaking mechanism of fragments in Model-Rect. At each iteration step fragments break into two pieces of equal mass such that the crack occurs perpendicular to one of the sides. After a fragment has been chosen for breaking, one of the three cases (*b*), (*c*), or (*d*) is selected with a probability depending on the length of the breaking edge.

**Figure 7:**
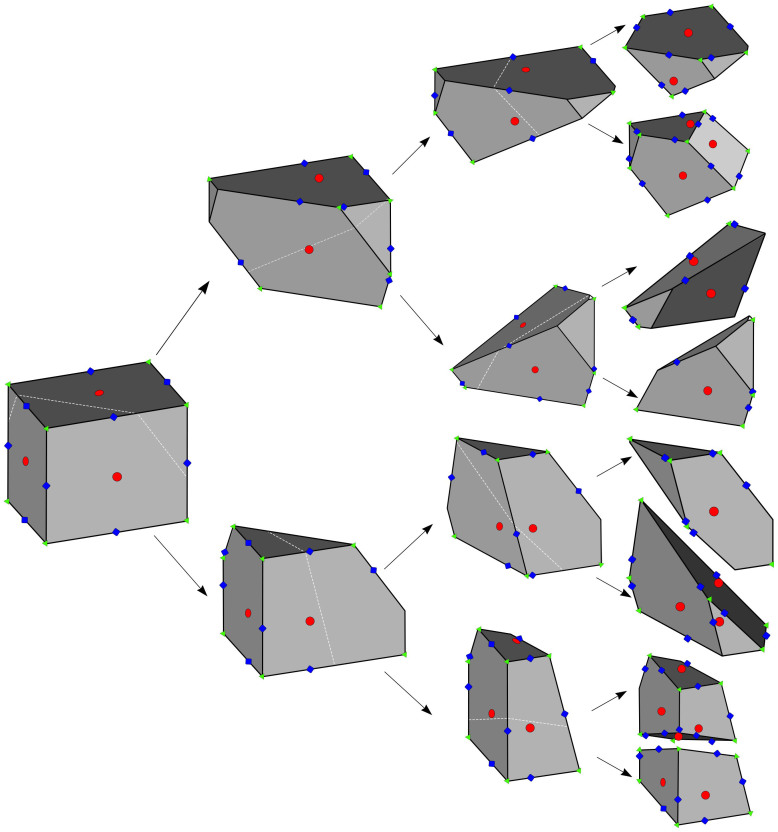
Breaking mechanism of the extended model (Model-Poly). Polyhedral fragments always break into two pieces along a crack plane which crosses the center of mass of the object with a random orientation. The figure presents the algorithm for three steps of the fragmentation process. Different types of equilibria are highlighted by different colors: red indicates stable points in the middle of the faces of the convex polyhedron, while green stands for the unstable points of the vertices. The blue dots in the middle of the edges are so called saddle points not discussed in the present work.

Figure 7 presents that starting from an initially cubic body this cracking process gives rise to fragments with convex polyhedral shapes very similar to real fragments. It can be observed in Figs. 2, 3, and 4 that the polyhedral extension of the discrete stochastic fragmentation model (Model-Poly) provides also a very good description of the experimentally obtained fragment mass distributions, shape-size relations, and of the statistics of different shapes, and simultaneously it also reproduces the statistics of stable and unstable points of fragments (see Fig. 5). Figure 7 also illustrates that the counting of different types of equilibria of fragments can be reduced to the identification of those faces and vertices of the polyhedra which carry stable and unstable equilibrium points, respectively. Two fundamental aspects of equilibrium points show a remarkable match with mathematical predictions: the *minimal value* is 2 both for *n_S_* and *n_U_*, as predicted by the *geometric robustness* of such shapes^46^ with respect to truncations. The expected values for both quantities are *below* the corresponding values of a cuboid *n_S_* = 6, *n_U_* = 8, confirming the prediction based on stochastic geometry^47^.

## Discussion

Fragmentation processes of heterogeneous brittle materials occur on widely different time scales having the weathering induced slow aging and the explosive breakup of rocks on the opposite limits. The governing cracking mechanisms are strongly different in the two cases, however, they both lead to fragments of polyhedral shapes with sharp edges. Our experiments demonstrated that in the investigated range of maximal size, both the mass and shape of fragments have an astonishing universality: the mass distribution of fragments proved to have a power law functional form with an exponential cutoff. The measured exponent is consistent with the branching-merging scenario of cracks in three dimensions which defines the universality class of these processes.

The size dependence of the shape of fragments exhibits a generic exponential convergence from the isotropic shape of small pieces to a uniquely defined anisotropic form for the large ones. Above a characteristic size the overall shape of all fragments can be well approximated by rectangles of dimensions 1 : 1.56 : 2.32. In nature fragmentation processes are the main source of fractured rocks which then undergo a long shape evolution due to weathering or collision induced abrasion e.g. in river beds. To obtain a comprehensive understanding of the observed shape of pebbles and boulders and their statistics, detailed knowledge is required on the initial configuration of the evolution^7^,^8^,^9^,^10^,^29^,^39^,^43^. A very important consequence of our results is that both for laboratory experiments and for modeling approaches of abrasion processes in the starting configuration fragments with power law distributed mass should be considered setting the overall aspect ratios according to our findings. Studying the statistics of stable and unstable equilibria of fragments we showed that beyond global geometrical features the complexity of polyhedral fragment shapes can also be grasped in a quantitative way. The range of validity of the scaling laws obtained in our study is given, on one hand, by the cutoff extension *L_c_* of fragments, on the other hand by the upper end *L* = 150 mm of the collected fragment range. Breakup processes producing finer-graded, or much larger fragments may be characterized by a higher degree of geometric complexity. The relevant features of materials considered in our study are the brittle mechanical response and the high degree of disorder at the mesoscopic scale which determine the universality class of fragment shapes.

Our experiments are limited to three-dimensional bulk solids. For the breakup of closed shells such as rocket fuel containers, relevant for the creation of space debris, self-affine shape of pieces has also been predicted^32^, i.e. larger fragments are more elongated, which is accompanied by a power law decay of the frequency of occurrence of different shapes of shell fragments. In our system no trace of self-affinity was found, and additionally, the statistics of fragment shapes proved to have an exponential decay. Comparing the threedimensional bulk solids to the locally two-dimensional shells demonstrates that the selection mechanism of fragment shapes is strongly affected by the interplay of the geometry of the fragmenting object and of the dimensionality of the embedding space.

We proposed a simple stochastic model of fragmentation which provides a very good quantitative description of all experimental findings including even the statistics of equilibrium points of fragments. The good agreement obtained under rather generic conditions implies the broad validity and impact of our results. In the model all geometrical features of fragments are controlled by a single parameter *α*, i.e. the power law exponent of the probability *p_c_* of how the crack plane orientation is selected depending on the linear extension of the body. In the realistic case of the polyhedral model *α* = 1 proved to be the physically relevant exponent, while other values of *α* merely compensate for the bias of how the linear extension is defined in terms of the fragment geometry.

## Methods

We carried out six rock fragmentation experiments under field conditions varying the type of fracturing, materials involved, and the geographical location. The experiments on geomaterials were complemented by laboratory measurements on gypsum. Table 1 summarizes the most important parameters of the experiments. Experiment A was performed by collecting fragments at the bottom of a cliff at Nagymaros city in Börzsöny mountain, Hungary. For experiments B and C fragments were generated by hammering the cliffs at the Navagio and Marathia beaches in Zakynthos, Greece, while for experiment D the same technique was used at Tündér-cliff, Budapest, Hungary. Dynamic fragmentation experiments (E and F in Table 1) were performed by exploding rock walls in the stone mines of Keszthely and Kádárta, Hungary. Gypsum cubes of size 150 mm × 150 mm × 150 mm were fragmented under laboratory conditions by manual hammering (experiment G). The fragmented materials were brittle rocks including dolomite, andesite, limestone, and a construction material gypsum all with a high degree of heterogeneity at the mesoscopic scale. In order to avoid systematic bias of sampling the fragment population, a well defined region of the fragmenting object was selected where all fragments were collected.

In order to characterize the shape of fragments the bounding box of single pieces was determined by means of a caliper. To minimize the error all fragments were treated by the same person executing the following procedure: side length *L* of the bounding box was obtained as the longest diameter of the fragment, then *I* is the largest distance perpendicular to the direction of *L*. Finally, side length *S* was defined as the largest distance perpendicular to the plane determined by *L* and *I*. A representative example of the bounding box is presented in Fig. 1. Stable and unstable equilibrium points of fragments were counted by hand putting the fragments on a hard table with various orientations. The counting technique has been presented in details in Ref. 44.

Computer simulations were carried out in the framework of our discrete stochastic models, Model-Rect and Model-Poly, of fragmentation. In order to keep the problem numerically tractable, the iterative process was stopped when degrading fragments reached a cutoff size. Quantities of interest such as the mass, size, and shape parameters of fragments were evaluated in an observation window which was set according to the size range of experiments. Good statistics of the simulation data was ensured by averaging over 1000 realizations of the breakup process. Model parameters such as the breaking probability *p* and the exponent *α* of the probability of crack plane orientation were determined by extensive simulations to obtain the best fit of the experimental results.

## Author Contributions

G.D., F.K., A.Á.S. and T.S. contributed equally to the work.

## Supplementary Material

Supplementary Information Supplementary information for G. Domokos, F. Kun, A. A. Sipos, and T. Szabó, *Universality of fragment shapes*

## Figures and Tables

**Table 1:** Most important parameters of the seven experiments we performed. The table provides the type of fragmentation, the materials involved, the number of fragments (Num. Frag.) obtained, and the size range of pieces in units of millimeter

Experiment	Type	Material	Num. Frag.	Size range [mm]
A	Weathering	Andete	295	20–120
B	Hammering	Limestone	336	18–147
C	Hammering	Limestone	468	20–185
D	Hammering	Dolomite	150	17–69
E	Explosion	Dolomite	1025	17–136
F	Explosion	Dolomite	317	16–131
G	Hammering	Gypsum	400	15–130
