# Supplementary material for: Universality of fragment shapes
Source: Sci Rep. 2015 Mar 16;5:9147. doi: 10.1038/srep09147 (PMC4360630; doi:10.1038/srep09147)
Supplement: Supplementary Information — for G. Domokos, F. Kun, A. A. Sipos, and T. Szabó, Universality of fragment shapes [file srep09147-s1.pdf]

Supplementary information for

G. Domokos[1], F. Kun[2*], A. Á. Sipos[1], and T. Szabó[1]

Universality of fragment shapes

[1]Department of Mechanics, Materials and Structures
Budapest University of Technology and Economics
Műegyetem rkp. 3., K242, 1111 Budapest, Hungary

[2]Department of Theoretical Physics, University of Debrecen
H-4010 Debrecen, P.O.Box: 5, Hungary
*Email: ferenc.kun@science.unideb.hu

Supplementary Figure S1 for the definition of stable and unstable equilibria

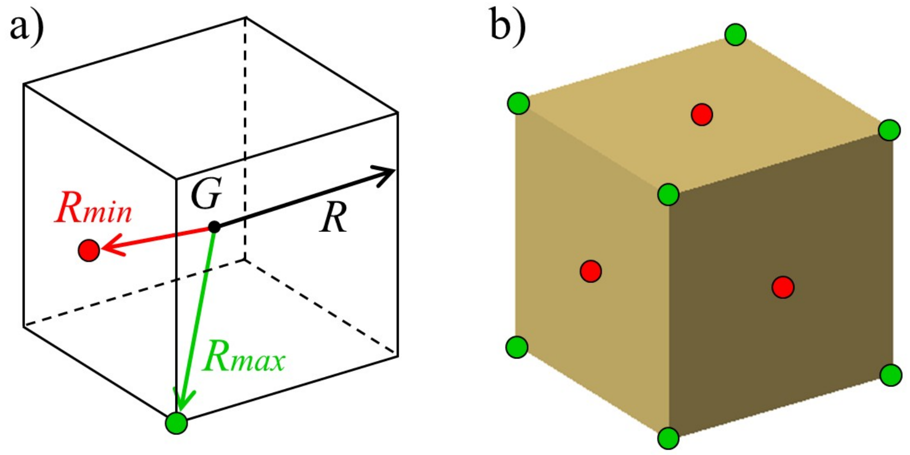

Figure S1: Definition of equilibrium points, illustrated on a cube. (a) Stable/unstable equilibrium points are the local minima/maxima of the distance function R measured from the center of gravity G. (b) On a cube, stable equilibrium points (red) are located at the middle of the 6 faces ($n_S = 6$), while unstable equilibrium points (green) are located at the 8 vertices ($n_S = 8$).

Supplementary information on the probability distribution of the number of stable and unstable equilibrium points

Although, the mass distribution of fragments is well understood, to the best of our knowledge, *nothing* has been published about the distributions of the number of stable n_S and unstable n_U equilibrium points of fragments. One can, however, make some simple observations based on earlier mathematical results [S1,S2]:

$$n_S, n_U \geq 2$$
$$E(n_S) \leq 6, \qquad E(n_U) \leq 8$$
$$\text{both densities } \; p(n_S) \text{ and } p(n_U) \text{ decay to zero at } n_S, n_U \to \infty.$$

Here E denotes the expected value of the stochastic variable. While there are infinitely many distributions fitting the above observations, the lognormal seems to be the first obvious choice. We also mention that there are partial results for the distribution of n_S and n_U on mature (well abraded) pebble populations: a Markov model suggests geometric distributions [S3]. However, the two cases (fragments and abraded particles) are fundamentally different. In Figure 5 of the manuscript we used the lognormal distribution only as a heuristic approximation of the data.

Supplementary information for Model-Rect

The sequential breakup model Model-Rect can be solved analytically for the mass distribution of fragments [S4]. Power law distribution is obtained

$$p(m) \sim m^{-\tau}, \tag{S1}$$

with an exponent τ depending on the value of the breaking probability p and on the number of fragments n created by the breakup of a single piece

$$\tau = \frac{\ln n}{\ln(1/p)}. \tag{S2}$$

In our model $n = 2$ daughter pieces are created, hence, the breaking probability $p \approx 0.665$ results in the exponent $\tau \approx 1.7$ obtained in the experiments. However, one has to note that in the analytic derivation an infinite breakup sequence is assumed without a lower cutoff. In our measurements and modeling a finite observation window of fragment sizes is considered which implies an upper and lower cutoff. This is the reason why the measured exponent

is reproduced by a higher value $p = 0.8$ of the breaking probability. The cutoffs, i.e. the size range of fragments are summarized in Table 1 of the manuscript.

Supplementary Figure S2 to illustrate the definition of the lists of length

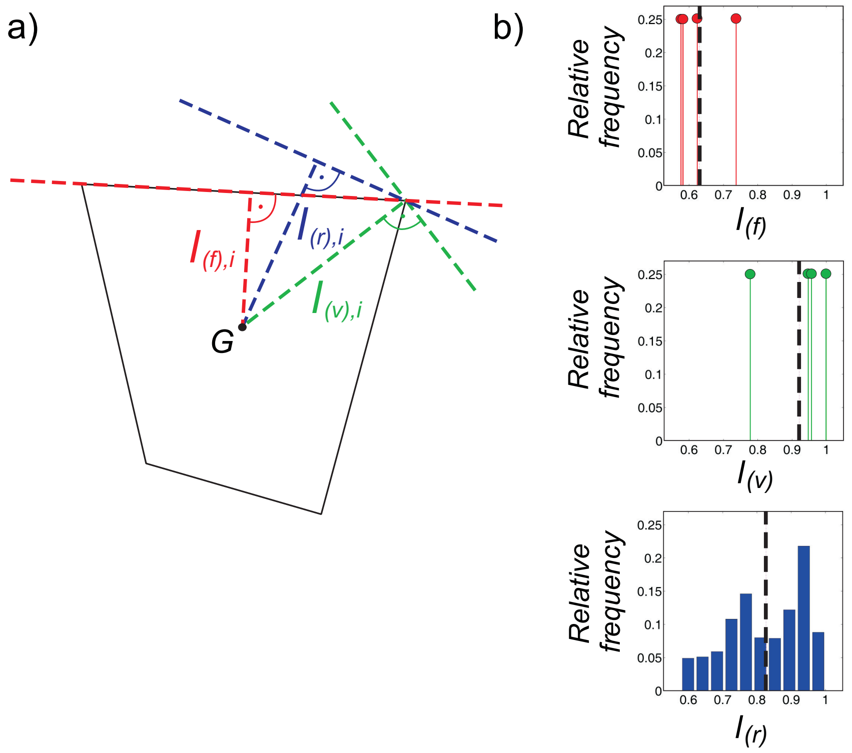

Figure S2: Illustration of the lists $l_{(f),i}$, $l_{(v),i}$ and $l_{(r),i}$ on a two-dimensional random polygon with vertices (-0.4495,-0.6351),(0.3615,-0.8760),(0.7558,0.5870),(-0.7498,0.6617). (a) Illustration of one random example for each of the three lists. (b) Histograms of $l_{(f)}$, $l_{(v)}$ and $l_{(r)}$. The histograms for $l_{(f)}$ and $l_{(v)}$ are based on the hypothesis that we sample uniformly from the lists $l_{(f),i}(i = 1, 2, \ldots F)$ and $l_{(v),i}(i = 1, 2, \ldots V)$, respectively. In the current example $F = V = 4$, so the relative frequency to pick any list-element is 0.25. The histogram for $l_{(r)}$ is based on randomly picking $N = 1000$ orientations $\underline{n}_i$ $(i = 1, 2, \ldots N)$ from a uniform distribution on the unit circle and calculating the distances to the corresponding N tangents. Bold black dashed vertical lines correspond to the mean values of $mean(l_{(f)})$=0.6297, $mean(l_{(v)}) = 0.9207$ and $mean(l_{(r)}) = 0.8261$, respectively.

References

[S1] Domokos, G., Lángi, Z. The robustness of equilibria on convex solids. *Mathematika* **60**, 237-256 (2014).

[S2] Schneider R., Weil W. *Stochastic and Integral Geometry* Springer-Verlag Berlin, Heidelberg (2008).

[S3] Domokos G., Monotonicity of Spatial Critical Points Evolving Under Curvature-Driven Flows. *J. Nonlin. Sci.* DOI: 10.1007/s00332-014-9228-3.

[S4] Turcotte, D.L. Fractals and Chaos in Geology and Geophysics. (Cambridge University Press, Cambridge, 1997).
